# Supplementary material for: The interactome of intact mitochondria by cross-linking mass spectrometry provides evidence for coexisting respiratory supercomplexes
Source: Mol Cell Proteomics. 2017 Dec 8;17(2):216–32. doi: 10.1074/mcp.RA117.000470 (PMC5795388; doi:10.1074/mcp.RA117.000470)
Supplement: Supplemental Data [file supp_RA117.000470_133922_0_supp_23978_fzffwf.zip › spectra_annotation/mito_spectra_annotation/301-1-2-1-3-1.pdf]

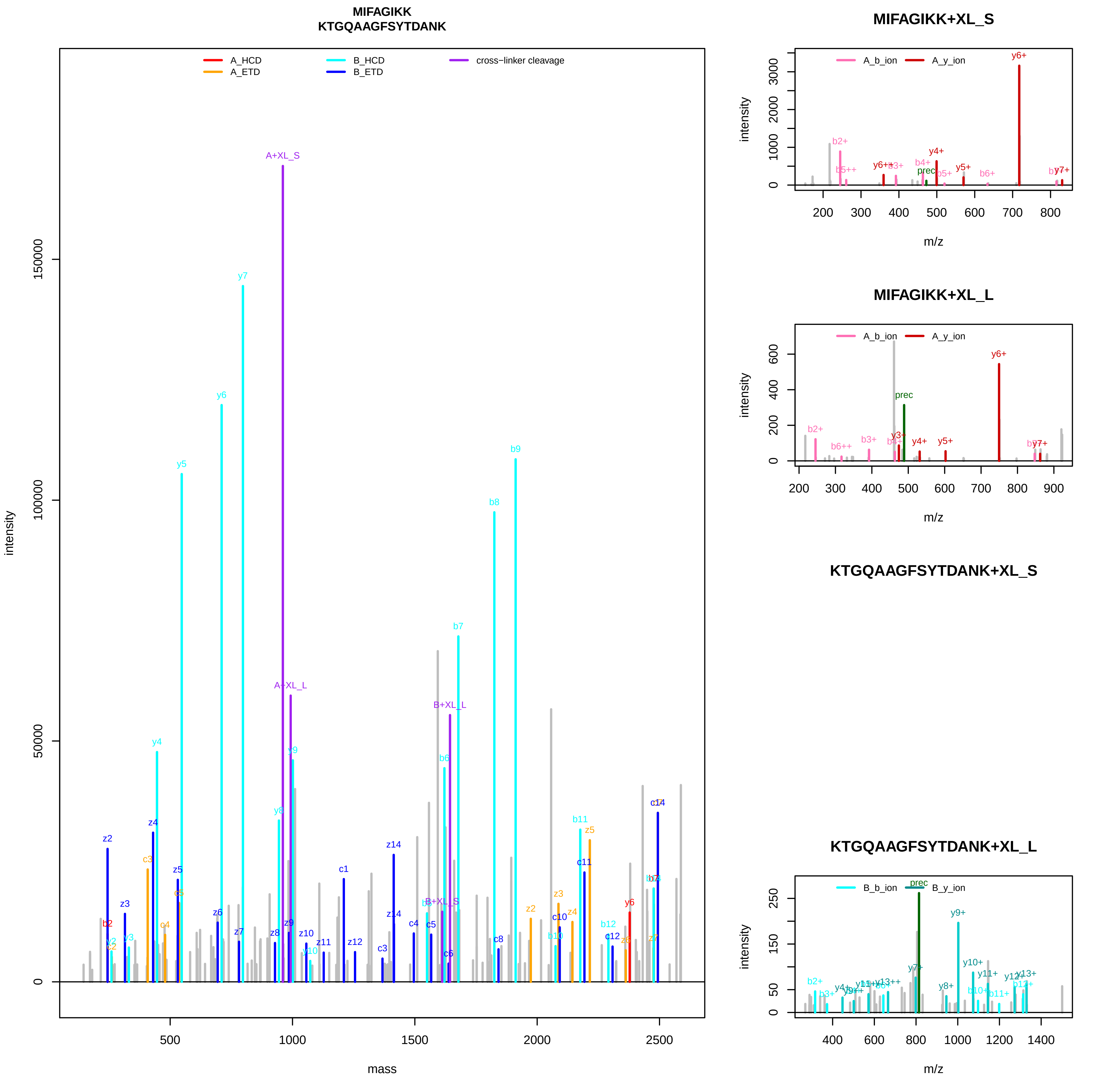

MIFAGIKK
KTGQAAGFSYTDANK
A_HCD
A_ETD
B_HCD
B_ETD
cross−linker cleavage
intensity
mass
0
50000
100000
150000
500
1000
1500
2000
2500
A+XL_S
A+XL_L
B+XL_L
B+XL_S
y4
y5
y6
y7
y8
y9
b6
b7
b8
b9
b11
b12
z2
z3
z4
z5
z6
z7
z8
z9
z10
z11
z12
z14
c1
c3
c4
c5
c6
c8
c10
c11
c12
c14
c3
c4
c5
z2
z3
z4
z5
b2
y6
MIFAGIKK+XL_S
A_b_ion
A_y_ion
intensity
m/z
0
1000
2000
3000
200
300
400
500
600
700
800
y6+
b2+
y4+
y5+
b6+
prec
MIFAGIKK+XL_L
A_b_ion
A_y_ion
intensity
m/z
0
200
400
600
200
300
400
500
600
700
800
900
y6+
prec
b2+
b3+
b6++
y3+
y4+
y5+
KTGQAAGFSYTDANK+XL_S
KTGQAAGFSYTDANK+XL_L
B_b_ion
B_y_ion
intensity
m/z
0
50
150
250
400
600
800
1000
1200
1400
prec
y9+
y10+
y11+
y7+
y8+
b2+
b3+
b10+
b11+
